# Analyzing possible intersections in the resistome among human, animal, and environment matrices

**DOI:** 10.3389/fmicb.2012.00418

**Published:** 2012-12-05

**Authors:** Stefania Stefani

**Affiliations:** Microbiology, University of CataniaCatania, Italy

The controversial question of microbial resistance origins, i.e., if it is the result of human activity or rather a synthesis of the evolution of antibiotic biosynthetic pathways that evolved over millions of years, is still challenging, with evidence for both. However, in the last few years, a growing body of evidence suggests a role of environmental microorganisms as reservoirs of resistance genes. The concept of the antibiotic resistome predicts that resistance is the result of a dynamic process involving microbial interactions in many different environments, all these occurring before the so called “antibiotic- era.” The fact that resistant microorganisms can explore a wide range of potential niches and acquire optimal adaptations for life in alternative hosts is worrisome, and could amplify their capability to acquire new determinants both in terms of virulence and resistance, with simultaneously maintaining their fitness. The variability of resistance determinants and their expression in different hosts are larger in nature than what is found in human pathogens, which implies the existence of bottlenecks modulating the transfer, spread, and stability of antibiotic resistance genes.

This research topic has collected the contributions of scientists involved in antibiotic resistance research, covering the current knowledge of single organisms and resistance determinants in various environments.

The first article, by Dr. Martínez (2012), analyzes the role of different factors that can affect the establishment of specific resistance determinants in a population of bacterial pathogens. These factors include founder effects, ecological connectivity, fitness costs, and second order selection. The Author then continues to cover housekeeping genes and human-driven contaminants.

The two papers that follow describe the possible interactions between animals and humans. The first study by Dr. Palmieri and co-authors (2011) describes the paradigmatic case of the major porcine pathogen *Streptococcus suis*, that is increasingly reported in severe infections in humans who come in contact with infected animal blood or secretions, or with pork-derived products. The available information on this microorganism, carrying many genetic elements similar to those identified in the major human pathogens *S. pyogenes* and *S. pneumoniae*, strongly suggests its role as a reservoir of resistance determinants for these important microorganisms. The second paper, by Dr. Pantosti (2012) which describes the case of livestock associated methicillin-resistant *Staphylococcus aureus* (LA-MRSA) is different. *S. aureus* is a typical human pathogen, and the recent findings that MRSA can colonize and evolve in animals, increase our concern that this MDR and virulent microorganism is able to adapt or readapt to humans and animals without losing fitness. Dr. Pantosti (2012) describes not only the first pig-MRSA, i.e., ST398, but also other animal-adapted MRSA clones, all detected in livestock, such as ST1, ST9, and ST130, this last carrying a new *mec*A gene.

The case of ubiquitous microorganisms such as enterococci, which are incredibly successful in adapting to different hosts and environments, increases the complexity of the role of the different evolutionary forces involved. This complex problem is reviewed by Dr. Santagati and co-authors (2012). The article evaluates the host-specific traits that are characteristic of some enterococcal species and addresses the presence of common and numerous mobile genetic elements that are important forces in evolution. These are spread over diverse hosts and environments, of at least the two major species studied, i.e., *E. faecalis* and *E. faecium*.

The double life of *Acinetobacter baumannii* as commensal and extremely successful pathogen is reviewed in depth in the paper by Dr. Roca and co-authors (2012). They exhaustively go through all the different biological aspects responsible for the success of these new MDR nosocomial pathogens, today almost untreatable with all common antimicrobial agents. Next to *Acinetobacter*, Dr. Pitout (2012) describes in his contribution the incredible evolution of extra-intestinal pathogenic *E. coli* (ExPEC): virulence genes acquired by horizontal gene transfer and the acquisition of a complex array of resistance determinants have made this species, and some epidemic MDR clones, the most worrisome microorganisms among Gram-negative species.

Two papers then address the evolution of mechanisms of resistance: Dr. Cantón and co-authors (2012), take into consideration one of the most paradigmatic mechanisms of resistance to beta-lactams, i.e., the CTX-M enzymes, while Dr. Poirel and co-authors (2012) look at the possible matrix intersection of plasmid mediated quinolone resistance. CTX-M, originally detected from the environmental *Kluvyera* spp., was successfully incorporated different times to originate different CTX-M clusters by the mobilization of specialized insertion sequences associated with a multifaceted genetic structure. Selective forces, including antibiotics, have fueled diversification and evolution of all these original clusters. Global spread was obtained after their uptake in epidemic resistance plasmids often harbored in high-risk epidemic clones. The ability to aggregate different resistance determinants (for example genes encoding different carbapenemases) makes the scenario of these pandemic MDR clones more complex.

The aquatic environment and farm animals seem to be the original source of plasmid mediated quinolone resistance, due to the action of different transferable mechanisms such as Qnr proteins, acetyltransferase AAC(6′)-Ib-cr and the efflux pumps QepA and QepB. Dr. Poirel, in the article, addresses the aquatic origin of the qnr genes.

The last two articles of this research topic are related to wildlife and bodies of water. Dr. Guenther and co-authors (2011) take the ESBL-producers *E. coli* into consideration, which was diagnosed in wild populations in Europe starting from 2006 and Dr. Lupo and co-authors (2012) review the most important mechanisms of resistance detected in water habitats and take into consideration the possible role of the bodies of water as matrices of reservoirs of resistance genes and of the spread of the mechanisms themselves.

In conclusion, MDR in all these pathogens is now widespread, resistance is pervasive and increasing in scope and impact. The antibiotic resistome has an enormous potential to provide new genes and new mechanisms; human use of antibiotics has provided the selective pressure necessary to capture, accommodate and make these complex structures functional, without affecting the bacterial fitness in diverse environments. Our knowledge on how antibiotics work and where resistance comes from is increasing: more has to be done, above all in natural environments, but for sure, resistance is natural, ancient and ineluctable.
